# Optimized protocol to preserve RNA integrity for laser capture microdissection of bovine mammary epithelial cells

**DOI:** 10.3168/jdsc.2024-0737

**Published:** 2025-04-11

**Authors:** Ratan Kumar Choudhary, Thomas B. McFadden, Erin M. Shangraw, Feng-Qi Zhao

**Affiliations:** 1 Department of Animal and Veterinary Sciences, University of Vermont, Burlington, VT 05405; 2 Division of Animal Sciences, University of Missouri, Columbia, MO 65211

## Abstract

Laser capture microdissection (LCM) is a popular technique for isolating specific cell types from tissues for medical research. However, the LCM procedure for isolating bovine mammary epithelial cells (MEC) for high-throughput transcriptome profiling has been lacking. The quality and quantity of RNA in LCM samples can be significantly affected by tissue treatment, time for dissection, and the total area of cells dissected. The objective of this study was to optimize the procedures for isolating bovine MEC from mammary tissues for consistent isolation of intact RNA suitable for downstream high-throughput RNA sequencing. Mammary tissues were biopsied (approximately 10–15 min per biopsy) from lactating cows, stored at −80°C, cryosectioned, and processed for LCM. Using LCM software, closed lines were selected and drawn around MEC visible within the alveoli of stained sections and microdissected. Degradation of MEC RNA was minimized by minimal exposure of tissue to aqueous media, the addition of RNase inhibitors in staining solution, and reducing LCM dissection time to less than 15 min (13.6 ± 0.52 min; mean ± SEM). Results showed that tissue fixation with chilled 70% ethanol, histone staining (with RNase inhibitor), dehydration in absolute ethanol, and final clearing in xylene preserved the RNA quality compared with staining sections without RNase inhibitor, no xylene clearing, and longer microdissection times. Using this approach, high-quality RNA was successfully obtained for RNA sequencing.

## Highlights

• We optimized staining and dehydration steps to minimize RNA degradation in stained tissue within 5 minutes.
• Precise LCM within 15 minutes further prevented RNA degradation.
• High-quality RNA isolated from MEC was suitable for RNA sequencing.

## Graphical Abstract

**Figure:**
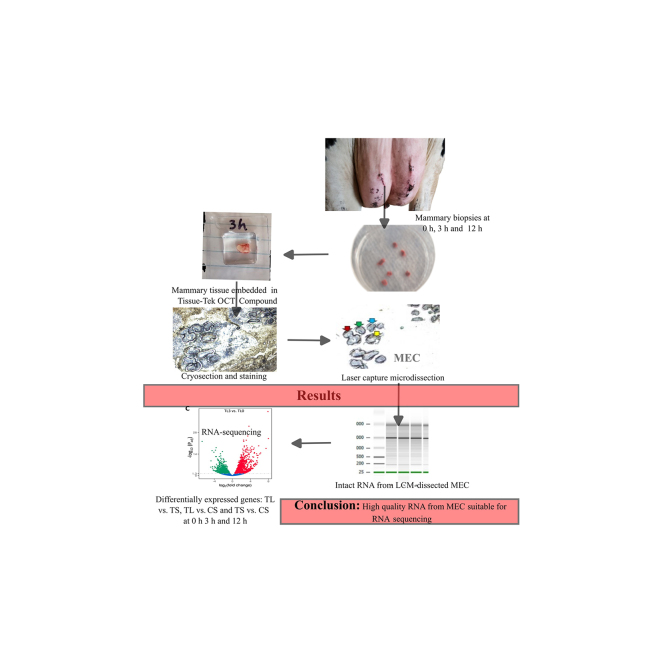
**Summary:** A laser capture microdissection (LCM) protocol was developed for whole-transcriptome profiling of lactating bovine mammary epithelial cells (MEC). Mammary tissue after the biopsy was embedded in optimal cutting temperature (OCT) compound and frozen in liquid nitrogen vapor for long-term storage at -80°C. For the effect of fixation time, a staining solution with and without RNase inhibitor was optimized for a good morphology of the stained section with minimum impact on cellular RNA integrity. Mammary alveolar epithelial cells were microdissected for total RNA isolation. The bioanalyzer measurements on RNA isolated from pooled microdissected MEC showed good-quality RNA suitable for RNA sequencing. The results of LCM sequencing revealed differentially expressed genes and transcriptomic signatures of bovine MEC.

Omics have become powerful high-throughput strategies to study physiological and pathological processes at the molecular level, where RNA sequencing (**RNA-seq**) is becoming the preferred method for global transcriptome and interactome activity (Islam et al., 2020; Hasankhani et al., 2023). Another cutting-edge methodology is laser capture microdissection (**LCM**), which stands out as a powerful technique that enables precise isolation of specific cell populations with the utmost precision from complex tissue samples. Laser capture microdissection allows in situ visualization of cells, counting of total objects (cells) dissected, and identification of cells with genetic labeling and positional information without tissue disruption (Rumpler et al., 2023). In the context of bovine mastitis and other mammary diseases, LCM allows the harvesting of pure mammary epithelial cells (**MEC**) directly from affected tissues (Choudhary et al., 2010). Capturing a pure population of MEC from mammary tissue and securing high-quality RNA from captured cells pose unique challenges, demanding stringent protocols for successful downstream analyses. The repeated mammary biopsy, tissue handling, strategy of tissue preservation with intact RNA, staining of tissue sections to visualize various cells of tissue vividly, acquisition of a sufficient number of pure populations of MEC, and optimal RNA integrity from harvested cells becomes particularly arduous. A streamlined workflow is imperative to ensure excised cells are promptly processed to mitigate potential RNA degradation. In addressing these challenges specific to MEC, a practical approach involving targeted LCM becomes crucial.

In this article, we present an optimized workflow for LCM that enables the reproducible isolation of high-quality RNA from MEC of normal or mastitic mammary tissue, facilitating robust transcriptomics analysis.

Mammary tissue samples were collected by biopsy to investigate the local and systemic effects of intramammary infusion of LPS in lactating cows (Shangraw et al., 2020, 2021; Choudhary et al., 2024). The procedures were approved by the Institutional Animal Care and Use Committees of the University of Missouri and the University of Vermont and carried out at the University of Missouri. The details of the animals and their management and treatment were described previously (Shangraw et al., 2020).

After morning milking, approximately 0.5 to 1.0 g of mammary tissue was removed using an drill-driven biopsy tool. Briefly, an approximately 10-cm^2^ area of skin on one rear quarter was clipped, shaved, and sterilized using 70% ethanol and iodine surgical scrub followed by local anesthesia using lidocaine hydrochloride. For each biopsy, a skin incision of 1 to 2 cm was made using a scalpel blade and an approximately 70 × 4 mm core of tissue was obtained using a biopsy tool as described elsewhere (Farr et al., 1996). Biopsies were taken from different sites on rear quarters at 0 h (before infusion), 3 h, and 12 h postinfusion. Each biopsy took less than 15 min. Within 5 min of collection, 30 mg of mammary tissue without visible stroma was diced into ∼1 × 1 mm^2^ pieces, quickly placed into a cryomold, and covered with specimen matrix (Tissue-Tek optimal cutting temperature [**OCT**] Compound, Sakura Finetek). The mold was then placed into the vapor phase above the liquid nitrogen vapor to snap freeze and stored in sterilized bags (Whirl-Pak) at −80°C for subsequent cryo-sectioning. All the working surfaces were first wiped with RNaseZap (Ambion) to remove possible contamination of RNase and then wiped with 70% ethanol. Tissue blocks were prepared to include samples from all 10 cows. Blocks were kept in a precooled cryostat at −20°C for at least 30 min before cutting 8-µm-thick sections. The knife holder was cleaned with 100% ethanol, and the brushes were treated with RNaseZap to eliminate RNase contamination. Next, the cryomold was removed and fixed onto the chuck (stainless steel holder) using OCT compound. The tissue holder containing the OCT-embedded tissue was placed on the cryochamber's metal grid and allowed to equilibrate to −20°C for at least 30 min. At least 5 cryosections were cut and deposited onto positively charged glass slides and immediately placed in a precooled slide box in a dry ice–containing thermocol box until final storage at −80°C. Before staining, sliding holding plastic jars (Pap-Fix Plus Empty Pap Jars) were appropriately cleaned to remove exogenous RNase, for which jars were first rinsed with 100% ethanol, then with distilled water, and followed by RNaseZap by inverting a few times and decanting. Finally, jars were rinsed 3 times with nuclease-free water and air-dried under a laminar flow hood. Before proceeding, forceps and tweezers were made RNase-free by wiping the surface with RNaseZap and nuclease-free 75% ethanol using lint-free tissue paper. Six jars (#1 to #6) were filled with following reagents: jar 1: 70% ethanol; jar 2: 95% ethanol; jar 3: 100% ethanol-1; jar 4: 100% ethanol-2; jar 5: xylene-1; and jar 6: xylene-2, respectively. A maximum of 4 tissue sections were processed at one time. Slides were removed from the deep freezer, thawed under the laminar flow for 30 s, and the back blotted with Kimwipes (Kimberly-Clark) to remove excess moisture. Using forceps, the slide was held at an angle, and chilled ethanol was added drop by drop onto the slides until the OCT compound loosened and became a flap. The flap of OCT compound was removed using forceps without touching tissue sections, and the sections were stained for 20 s with 100 µL of Histogene (Thermo Fisher Scientific) solution premixed with RNase inhibitor at the concentration of 1 U/µL (to 100 µL of stain solution, 2.5 µL of RNasin Plus RNase Inhibitor [Promega] was added). After tilting the slides on tissue paper to drain the excess stain solution, slides were dipped in 75% ethanol (jar 1; 20 s) to remove excess stain, followed by dehydration in a series of 95% ethanol (jar 2; 20 s) and 2 changes of absolute ethanol (jar 3 and 4; 30 s each). Finally, slides were cleared in the first xylene (jar 5; 1 min) and second xylene (jar 6; 2 min). One slide from the second xylene solution was taken out, placed under the fume hood for drying (5 min), then used for LCM. Other slides remained in the second xylene until microdissection of each preceding slide was completed. Slides remained in xylene for 4 to 5 h without damaging RNA quality of enteric ganglia cells (May-Zhang et al., 2018) or bovine mammary tissue sections (Choudhary et al., 2013). Histogene-stained MEC had distinct epithelial and stromal areas outlined using an LCM microscope at 100× magnification (Figure 1A). Boundaries of alveolar epithelial cells were marked distinctly and underwent LCM as described below. Thus, stromal cells were excluded from the dissection (Figure 1B).

**Figure 1:**
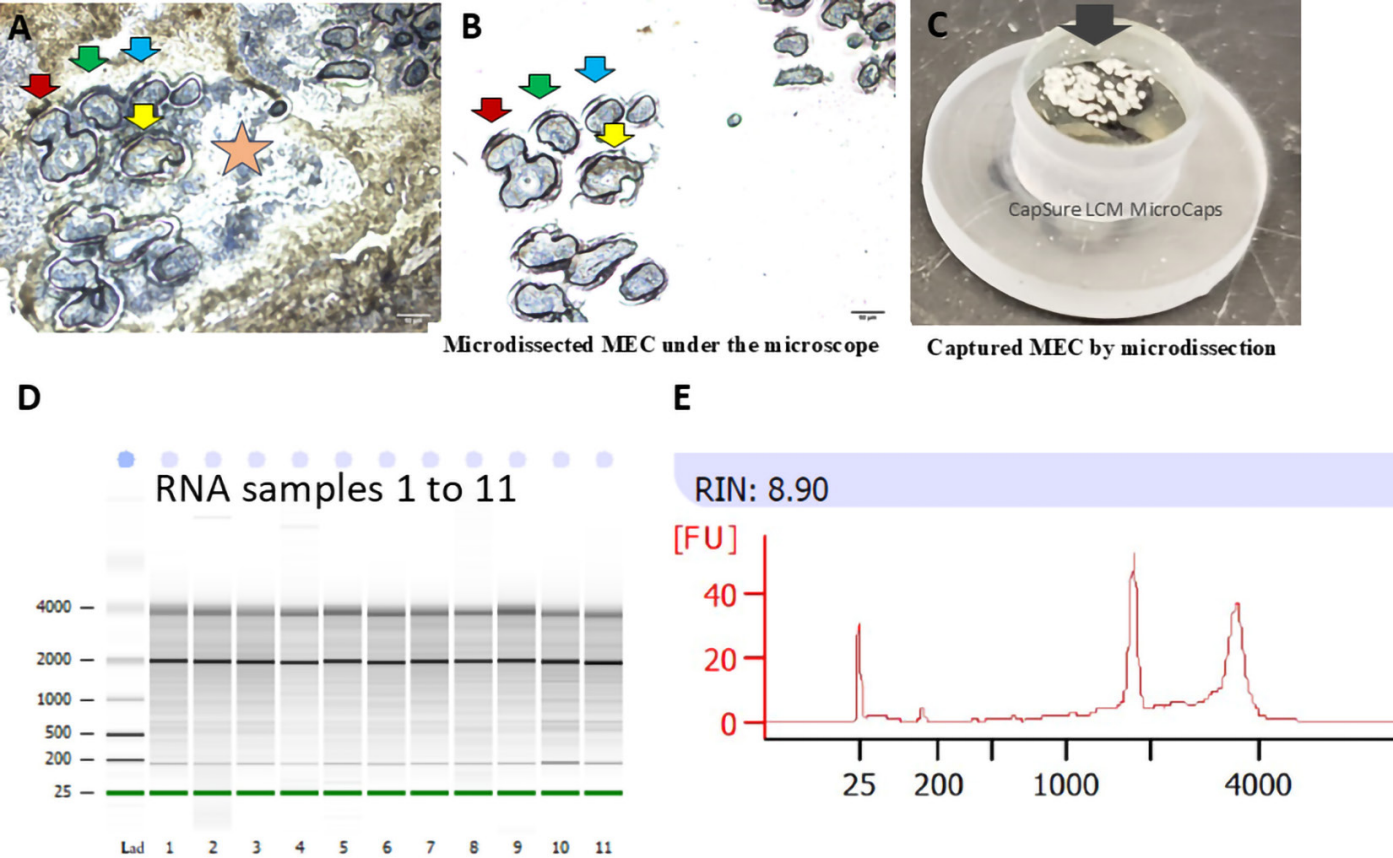
A representative image of histogene-stained mammary epithelial cells (MEC) in lactating bovine mammary tissue, laser capture microdissection sections, and RNA quality. (A) A mammary tissue section was stained and visualized (100×), and multiple paths for dissection were drawn. Length of scale bar is 50 µm. Red, green, blue, and yellow arrows represent individual alveoli surrounded by MEC, and the star represents the stromal cells. Stromal cells were not dissected. (B) Corresponding microdissected alveoli indicated by red, green, blue, and yellow arrows in panel A are visible on the cap of the collector tube (10×) without the contamination of stromal cells. (C) Image of groups of microdissected cells in collector cap visualized under the microscope. (D) Electrophoresis image of total RNA isolated from microdissected MEC by the Agilent BioAnalyzer. (E) A representative image of the RNA integrity number (RIN) of one total RNA sample from capillary electrophoresis of the BioAnalyzer electropherogram. [FU] = fluorescent units; Lad = ladder.

The laser capture microdissection procedures described herein are based on the Arcturus LCM system (ThermoFisher Scientific). The whole procedure for staining and microdissection was performed at the Vermont Integrative Genomics Resource DNA Facility of the University of Vermont (Burlington, VT). The LCM staging area and contacting surfaces, including the table area used during the operation, were treated with RNaseZap and 70% ethanol. Upon calibrating the laser on the path of the specimen area under selection, the objective 10× was chosen along with the respective dissected cells collecting cap (CapSure Macro LCM caps, ThermoFisher). Using line tools, closed lines were selected and multiple shapes were drawn around the MEC of the alveoli visualized in the microscopic field. After the selection of areas of MEC, the power, aperture, and speed of the laser were set to 50 mW, f20, and 10 µm/s, respectively. Then, “start cut” was selected to dissect MEC from selected areas using laser, and the procedures were repeated with another microscopic field. Laser-cutting operations continued unless all MEC of the slide were cut. Immediately after dissection, CapSure Macro LCM caps (Figure 1C) were secured onto 0.5-mL microfuge tubes filled with 230 μL of RLT buffer, a component of RNA isolation kit (RNeasy Plus Micro Kit; catalog no. 74034; Qiagen) premixed with β-mercaptoethanol. Each tube was kept inverted, then incubated at room temperature for 30 min for complete cell lysis. Finally, each tube was centrifuged (5,000 × *g*; 5 min at room temperature), frozen on dry ice, and stored at −80°C until RNA isolation.

Cell lysates were mixed with an equal volume of 70% ethanol and processed for total RNA isolation using the RNeasy Micro Kit from Qiagen. The quality and integrity of total RNA extracted from dissected LCM samples were appraised via RNA integrity number (**RIN**), measured by the electrophoretic RNA analysis, and expressed on a scale of 1 (degraded) to 10 (intact). Aliquots of each RNA sample were also analyzed on a Bioanalyzer RNA 6000 Pico chip (Agilent Technologies) using the 2100 BioAnalyzer (Agilent Technologies; Figure 1D and 1E).

The major steps optimized for the seamless collection of high-quality RNA for downstream application involved mammary tissue collection, LCM, and RNA isolation. Mammary tissues snap-frozen in the OCT compound were processed on the spot at the time of collection before samples were brought to the laboratory. All the staining jars were kept nuclease-free, and an RNase inhibitor was added to the staining solution to minimize degradation of RNA. Use of the Histogene staining solution containing cresyl violet stain with our optimized quick staining protocol (elapsed time from staining through mid-dissection less than 30 min) resulted in good morphological resolution of the tissue samples and yielded intact RNA with a high RIN. Tissue blocks and cut sections were stored at −80°C in a freezer and cut once rather than repeatedly thawing, sectioning, and conducting microdissection. The short distance between the hood under which cryosections were stained before LCM and the microdissection microscope in an adjacent room helped minimize the time between staining and LCM dissection.

To optimize our procedures, different steps, including staining of cryosections, with chilled fixation, histostaining, and short duration of staining coupled with washings were tested multiple times to minimize RNA degradation. Upon achieving an acceptable RIN in RNA isolated from tissue lysates, sections were then used for microdissection. High-quality RNA, as indicated by acceptable RIN values, is crucial for downstream applications. This was confirmed by assessing RNA samples via Bioanalyzers before shipping RNA samples for sequencing and again after receiving RNA samples at the sequencing facility. This method generates a numerical RIN score, with 10 representing intact RNA and 1 indicating severe degradation. In our analysis of 60 LCM samples, the average RIN was 6.22 ± 1.16. Of 45 samples dispatched for transcriptomic services, RNA quality was tested again at the sequencing facility, and the average RIN for 45 samples sequenced was 7.16 ± 1.68, suggesting good RNA quality and suitability for deep sequencing.

Whole mammary tissues are typically used in the conventional technique of RNA-seq while analyzing the impact of mastitis-causing pathogens (Hasankhani et al., 2023) or LPS (Shangraw et al., 2021). Likewise, RNA-seq analysis of primary culture of bovine MEC challenged with a pathogen like *Staphylococcus aureus* (Wang et al., 2020) may not represent the actual changes occurring in MEC in situ. The LCM offers a powerful technique for isolating pure populations of cells directly from their native tissue context. This stands in contrast to flow cytometry, where enzymatic tissue dissociation generates a single-cell suspension, sacrificing valuable spatial information and potentially compromising cell identity and purity. Laser capture microdissection allows researchers to target specific cells, including immunolabeled MEC, and isolate them from adjacent unlabeled cells for their gene expression profiles (Choudhary et al., 2013). This opens doors for studying disease progression, cell-cell interactions, cell characterization, including tissue-specific stem cells, and tissue heterogeneity with unparalleled precision.

Isolation of intact RNA from LCM is susceptible to degradation due to various processing steps of tissue sectioning, staining, and microdissection, resulting in a wide range of RIN values of the RNA ranging from 4 to 9 (Cánovas et al., 2014). One of the main reasons that cause RNA degradation during LCM is the endogenous RNase activity in the tissue rendered active by aqueous solution (Clément-Ziza et al., 2008). The selection of tissue fixatives, minimal washing steps using an aqueous solution, and the addition of RNase inhibitors facilitate the recovery of intact RNA from microdissected MEC (Choudhary et al., 2010). Reports suggest that RNA quality affects gene expression studies; thus, discrepancies can arise depending on the quality of mammary RNA (Eisenberger et al., 2004; Cox et al., 2008).

In summary, we described a robust and efficient LCM protocol suitable for high-quality RNA extraction from microdissected bovine MEC and validated the workflow through RNA-seq. By combining the precision of LCM with the power of RNA-seq, researchers can gain unprecedented access to the physiology and pathology of specific cell populations within tissues. This opens doors to a deeper understanding of complex biological processes in understanding of bovine mastitis at the cellular level.
